# Investigating Causal Relationships Between Psychiatric Traits and Intracranial Aneurysms: A Bi-directional Two-Sample Mendelian Randomization Study

**DOI:** 10.3389/fgene.2021.741429

**Published:** 2021-10-19

**Authors:** Peng Peng, Zirong Chen, Xiaolin Zhang, Zhongyin Guo, Fangyong Dong, Yu Xu, Yue He, Dongsheng Guo, Feng Wan

**Affiliations:** Department of Neurosurgery, Tongji Hospital of Tongji Medical College, Huazhong University of Science and Technology, Wuhan, China

**Keywords:** psychiatric traits, intracranial aneurysms, mendelian randomization, causality, GWAS summary statistics

## Abstract

**Background** Despite psychiatric traits were associated with intracranial aneurysms (IAs) in observational studies, their causal relationships remain largely undefined. We aimed to assess the causality between psychiatric traits and IAs.

**Methods** We firstly collected the genome-wide association statistics of IAs (sample size, *n* = 79,429) and ten psychiatric traits from Europeans, including insomnia (*n* = 1,331,010), mood instability (*n* = 363,705), anxiety disorder (*n* = 83,566), major depressive disorder (MDD) (*n* = 480,359), subjective wellbeing (*n* = 388,538), attention deficit/hyperactivity disorder (ADHD) (*n* = 53,293), autism spectrum disorder (ASD) (*n* = 46,350), bipolar disorder (BIP) (*n* = 51,710), schizophrenia (SCZ) (*n* = 105,318), and neuroticism (*n* = 168,105). We then conducted a series of bi-directional two-sample Mendelian randomization (MR) analyses, of which the Robust Adjusted Profile Score (RAPS) was the primary method to estimate the causal effects between these psychiatric traits and IAs.

**Results** We found that insomnia exhibited a significant risk effect on IAs with the odds ratio (OR) being 1.22 (95% CI: 1.11–1.34, *p* = 4.61 × 10^–5^) from the RAPS method. There was suggestive evidence for risk effect of mood instability on IAs (RAPS, OR = 4.16, 95% CI: 1.02–17.00, *p* = 0.047). However, no clear evidence of causal effects on IAs for the rest eight psychiatric traits (anxiety disorder, MDD, subjective wellbeing, ADHD, ASD, BIP, SCZ, and neuroticism) was identified. In the reverse MR analyses, no causal effects of IAs on psychiatric traits were found.

**Conclusions** Our findings provide strong evidence for a causal risk effect of insomnia on IAs and suggestive evidence for mood instability as a causal risk effect on IAs. These results could inform the prevention and clinical intervention of IAs.

## Introduction

Intracranial aneurysms (IAs) are widespread life-threatening diseases (Vlak et al., 2011). Rupture of IAs results in aneurysmal subarachnoid hemorrhage (aSAH), a form of stroke that accounts for 5% of all strokes. Half of aSAH patients are younger than 55 years and a third of the patients die within the first few days to weeks of bleeding. Most survivors suffer from long-term disability or cognitive impairment (Nieuwkamp et al., 2009).

Among patients with IAs, psychiatric traits usually co-exist. Observational studies revealed IAs and psychiatric traits like insomnia (Colledge et al., 2017; Zhang et al., 2020), mood instability (McGowan et al., 2021), anxiety disorder (Lambiase et al., 2014; Lemos et al., 2020; Zhai et al., 2020), major depressive disorder (MDD) (Henderson et al., 2013; Tang et al., 2020; Zhai et al., 2020), subjective wellbeing (Tabernero et al., 2019), attention deficit/hyperactivity disorder (ADHD) (Jeong et al., 2015), autism spectrum disorder (ASD), bipolar disorder (BIP) (Prieto et al., 2014), schizophrenia (SCZ) (Liang et al., 2016), and neuroticism (Marijnissen et al., 2014) were bidirectionally correlated. For example, Marijnissen et al. found depression was a risk factor for stroke, including IAs, via a 9-year cohort study (Marijnissen et al., 2014). Inversely, among the 200 unruptured IAs treated by endovascular intervention, 31 (15.5%) had depression and 34 (17.0%) had anxiety after discharge (Zhai et al., 2020). Another example, a meta-analysis revealed insomnia might increase the risk of future IAs formation (He et al., 2017), while the reverse relationship between insomnia and IAs was also reported (Colledge et al., 2017). However, there was insufficient evidence on whether these psychiatric traits causally lead to IAs or vice versa, owing to possible residual confounding and reverse causation bias in observational researches (van’t Hof et al., 2017).

Causal inference between diseases and complex traits could be conducted under the development of Mendelian randomization (MR) and genome-wide association studies (GWAS). MR is a causal inference method for estimating the modifiable exposure (for example, insomnia) to an outcome (for example, IAs) via taking genetic variants as instrumental variables (IVs) for the exposure (Davey Smith and Ebrahim, 2003). MR method decreases residual confounding since the IVs are initially realigned in a random manner without connection to environmental factors, behaviors, and self-selected lifestyle factors. Besides, the MR method conquers reverse causality since IVs are determined irrespective of disease progression or development. Here, we conducted bi-directional two-sample MR analyses using GWAS summary data of IAs and the ten above-mentioned psychiatric traits to infer their causality. Clarifying such causal relationships from the genetic perspective may have important implications for primary prevention strategies in IA patients.

## Materials and Methods

### Data Extraction

Summary data from GWAS for IAs and eight psychiatric traits was gathered from released researches with the biggest European population sample (Table 1). For restriction, only summary data of SNPs that were significantly related to mood instability and anxiety disorders (the other two psychiatric traits) was obtained. GWAS of IAs were generated from the stage 1 association study of European ancestry, consisting of 7,495 cases and 71,934 controls passing quality control thresholds. The definitions of the ten psychiatric traits were laid out in Supplementary Table S1.

**TABLE 1:** Details of GWAS phenotypes utilized for each trait.

Trait	Cases, no	Controls, no	Sample overlap	Consortium	Data source
Intracranial aneurysms	7,495	71,934	—	Europeans	@neurIST, ARIC, Busselton, Utrecht 1, Netherlands (EGA), Utrecht 2, Doetinchem Cohort Study, Project MinE, French Canadia, Finland (EGA), Finland, NFBC1966, ICAN, PREGO, GAIN, FIA, nonGAIN, Poland, NBS, UKB, GOSH controls, GOSH cases, NBS+1958BBC
Insomnia	397,972	933,038	1.0%	Europeans	UKB, 23andMe
Mood instability	157,039	206,666	3.7%	Europeans	UKB
Anxiety disorders	25,453	58,113	16.3%	Europeans	UKB
MDD	135,458	344,901	2.8%	Europeans	UKB, 23andMe, PGC29, deCODE
					GenScot, GERA, iPSYCH
Subjective wellbeing	388,538*^a^*	388,538*^a^*	3.5%	Europeans	UKB, 23andMe, SSGAC
ADHD	19,099	34,194	0	96% of Europeans	PGC
ASD	18,381	27,969	0	Europeans	PGC
BIP	20,352	31,358	0	Europeans	PGC
SCZ	40,675	64,643	0	Europeans	CLOZUK, PGC
Neuroticism	168,105*^a^*	168,105*^a^*	8.1%	Europeans	UKB, GPC

ARIC, The Atherosclerosis Risk in Communities; NFBC1966, the Northern Finnish Birth Cohort 1966; ICAN, Intracranial aneurysm; PREGO, the Population de Référence du Grand Ouest biobank; GAIN, the Genetic Association Information Network study; FIA, the Familial Intracranial Aneurysm cohort; NBS, National blood donors; UKB, the United Kingdom Biobank; GOSH, Genetics and Observational Subarachnoid Haemorrhage Study; 1958BBC, 1958 British Birth cohort; 23andMe, 23andMe company; MDD, major depressive disorder; PGC29, the Psychiatric Genomics Consortium, 29 European samples; deCODE, deCODE Genetics company; GenScot, Generation Scotland: Scottish Family Health Study; GERA, Genetic Epidemiology Research on Adult Health and Aging Study; iPSYCH, The Lundbeck Foundation Initiative for Integrative Psychiatric Research; SSGAC, Social Science Genetics Association Consortium; ADHD, attention defificit/hyperactivity disorder; PGC, the Psychiatric Genomics Consortium; ASD, autism spectrum disorder; BIP, bipolar disorder; SCZ, Schizophrenia; GPC, the Genetics of Personality Consortium.

a Number of the total sample size.

The overlapping sample size is divided by the larger sample size of intracranial aneurysms and the corresponding psychiatric trait.

### IVs Selection and Bi-Directional MR Analyses

**FIGURE 1:**
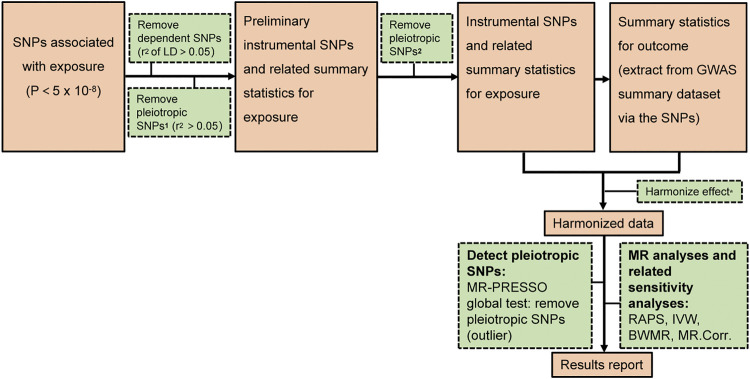
The analysis flowchart of this study. ^**1**^ the possible pleiotropic SNPs which are associated with significant SNPs of outcome. ^2^ the possible pleiotropic SNPs which are associated with outcome (below the genome wide suggestive significant level of 10^−5^) Harmonize effect^*^: 1 Ensure that the SNPs’ effect on the exposure and outcome correlate to the same allele 2 Remove SNPs with inconsistent alleles between the exposure and outcome.

Figure 1 depicted our research workflow. The following documented valid IVs were selected based on the suppositions of MR. First, SNPs that under the threshold (*p <* 5 × 10^–8^) and associated with the exposure were selected as candidate IVs. Second, We used linkage disequilibrium (LD) (Chang et al., 2015) to further exclude dependent SNPs. Third, the significant SNPs in LD (*r* ^2^ *>* 0.05) were filtered out based on bi-directional MR requirements (no LD or overlap in the IVs between the exposure and outcome) (Davey Smith and Hemani, 2014). Fourth, potentially pleiotropic SNPs were removed by excluding those with suggestive association with IAs (*p <* 10^–5^) (Bakker et al., 2020). The rest SNPs were defined as valid IVs for MR analyses. Supplementary Tables S2–S19 showed the valid IVs in this study. The *F* statistics (Lawlor et al., 2008) and the 95% confidence interval (*CI*) (Burgess et al., 2016) were computed to quantify whether the IVs were strongly related to the exposure. We set the *p* < 0.005 (0.05/10) for multiple comparisons to test each result to avoid false positive results.

Here, we used four different methods, specifically the Robust Adjusted Profile Score (RAPS) method, the Inverse Variance Weighted (IVW) method, the Bayesian Weighted Mendelian Randomization (BWMR), and the MR (MR.Corr) method. Compared with the other three methods, the RAPS method is more efficient in dealing with residual horizontal pleiotropy and outliers, thereby is chosen as the primary method (Zhao et al., 2018; Qi and Chatterjee, 2021). RAPS is designed to conduct two-sample MR analysis with summary statistics by Zhao etc (Zhao et al., 2018). We applied the RAPS method in sensitivity analyses to confirm MR presumptions and control for overdispersion as an indicator for systematic pleiotropy. The IVW method is based on the following assumptions: the overall bias is zero, or all SNPs are valid IVs (Bowden et al., 2016). Potential heterogeneity, measured by Cochran’s Q statistic, was evaluated via multiplicative random effects IVW (Hemani et al., 2018). BWMR method could efficiently investigate the causal effect between the exposure and outcome via the standard error and *p*-value based on GWAS summary statistics (Zhao et al., 2020). MR.Corr is a method employing correlated instrumental variants explaining correlated horizontal pleiotropy during two-sample MR (Cheng et al., 2020). All statistics analyses were executed with two-sample MR and MR-PRESSO packages in R version 3.5.3.

## Results

### Causal Effects of Psychiatric Traits on IAs

**FIGURE 2:**
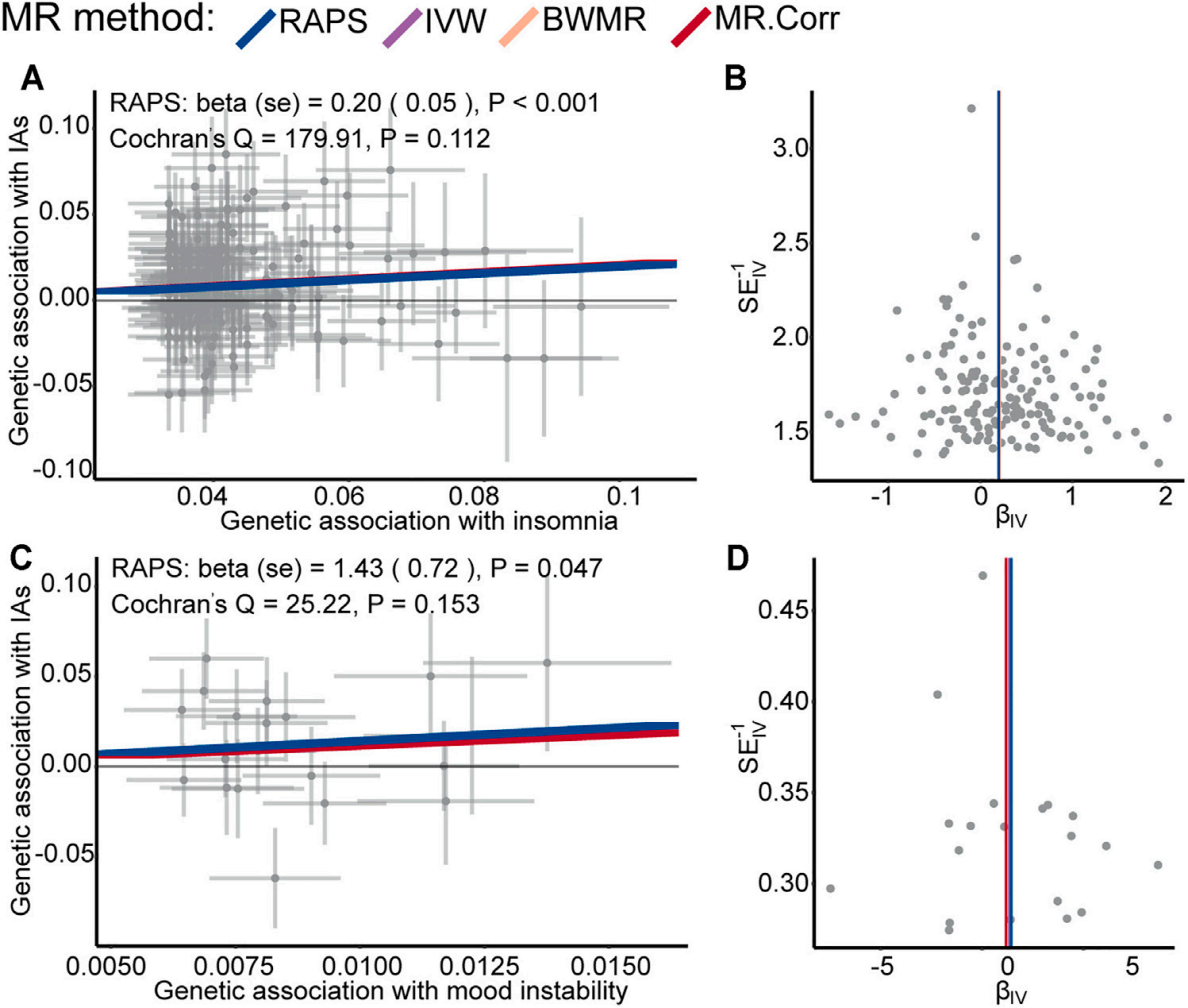
Scatter plots of SNPs with IAs versus. SNPs with insomnia **(A)** and mood instability **(C)** for all the valid IVs. Each dot represents one SNP, with corresponding standard error bars of its relation to insomnia, mood instability (*y*-axis) and IAs (*x*-axis); the colored solid lines represent estimated causal effect values of four MR methods. Funnel plots of the causal effect point estimate about insomnia **(B)** and mood instability **(D)** on IAs displaying a symmetric shape; The vertical colored lines represent the estimated causal effect acquired utilizing all IVs.

The valid SNPs with IAs over that with psychiatric traits were displayed in scatter plots of Figures 2A,C; Supplementary Figure S1. After removing outlier SNPs via the MR-PRESSO outlier test, all four MR approaches approved well in fitting the linear relation between the SNPs effect on IAs and the exposures. The estimated causal effects of the ten exposures on IAs were listed in Supplementary Table S2. The result showed insomnia exhibited a significant risk effect on IAs with the odds ratio (OR) being 1.22 (95% CI: 1.11–1.34, *p* = 4.61 × 10^–5^) from RAPS, and similar risk estimates were obtained using the other three methods (IVW, OR = 1.22, 95% CI :1.11–1.34, *p* = 5.14 × 10^–5^, BWMR, OR = 1.23, 95% CI :1.11–1.36, *p* = 4.87 × 10^–5^, MR.Corr, OR = 1.23, 95% CI :1.11–1.35, *p* = 6.22 × 10^–5^, Figure 2A and Table 2). The result of RAPS method suggested mood instability had a risk effect on IAs (OR = 4.16, 95% CI: 1.02,17.00, *p* = 0.047, 0.005 < *p* < 0.05) (Figure 2C). Because the four colored solid lines merged together in Figures 2A,C, the results of the four MR methods were shown separately in Supplementary Figures S2, S3. Similar ORs from IVW, BWMR, MR.Corr methods were 3.83 (95% CI: 0.82–17.88), 3.84 (95% CI: 0.77–19.12), 3.22 (95% CI: 0.84–12.35), respectively, though they were not statistically significant (Table 2). Furthermore, funnel plots of the causal effect point estimate about insomnia and mood instability on IAs displaying a symmetric shape (Figures 2B,D). However, no genetic evidence of causal effects on IAs for the rest eight psychiatric traits (anxiety disorder, MDD, subjective wellbeing, ADHD, ASD, BIP, SCZ, and neuroticism) was identified (Supplementary Figure S1 **)**. Null of our IVs were subject to weak instrument bias, as each *F* statistics was not less than 32 (Table 2 **)**. Importantly, Cochran’s Q-test revealed no pleiotropic effect or horizontal heterogeneity.

**TABLE 2:** The causal effects of the ten psychiatric traits on IAs by two-sample MR analyses.

Exposure	N SNPs	MR methods								*F* Statistics
		RAPS		IVW		BWMR		MR.Corr		
		*OR* (95% *CI*)	*p*	*OR* (95% *CI*)	*p*	*OR* (95% *CI*)	*p*	*OR* (95% *CI*)	*p*	
Insomnia	159	**1.22** (1.11,1.34)	**4.61 × 10** ^**–5**^	**1.22** (1.11,1.34)	**5.14 × 10** ^**–5**^	**1.23** (1.11,1.36)	**4.87 × 10** ^**–5**^	**1.23** (1.11,1.35)	**6.22 × 10** ^**–5**^	41.47 (39.41,43.87)
Mood instability	20	**4.16** (1.02,17.00)	**0.047**	**3.83** (0.82,17.88)	0.087	**3.84** (0.77,19.12)	0.101	**3.22** (0.84,12.35)	0.088	39.20 (35.70,43.07)
Anxiety disorder	5	1.07 (0.90,1.27)	0.447	1.08 (0.91,1.29)	0.388	1.08 (0.89,1.31)	0.431	1.10 (0.93,1.29)	0.279	34.69 (30.77,39.70)
MDD	26	1.23 (0.92,1.65)	0.165	1.22 (0.93,1.61)	0.153	1.23 (0.92,1.63)	0.156	1.23 (0.95,1.59)	0.121	38.41 (35.33,42.00)
Subjective wellbeing	32	0.55 (0.26,1.14)	0.11	0.59 (0.29,1.17)	0.128	0.58 (0.29,1.17)	0.129	0.57 (0.27,1.20)	0.138	36.70 (34.67,38.92)
ADHD	9	1.15 (0.94,1.41)	0.166	1.15 (0.95,1.39)	0.144	1.16 (0.95,1.41)	0.143	1.11 (0.92,1.48)	0.206	32.38 (28.22,37.81)
ASD	4	0.93 (0.60,1.42)	0.721	0.93 (0.62,1.38)	0.705	0.92 (0.61,1.40)	0.71	0.92 (0.61,1.40)	0.692	38.44 (32.21,47.39)
BIP	13	0.90 (0.75,1.09)	0.301	0.87 (0.72,1.04)	0.134	0.86 (0.71,1.05)	0.138	0.88 (0.72,1.07)	0.194	34.84 (32.39,37.81)
SCZ	76	1.02 (0.94,1.11)	0.621	1.03 (0.94,1.12)	0.542	1.03 (0.94,1.12)	0.533	1.03 (0.95,1.12)	0.529	41.66 (39.22,44.30)
Neuroticism	19	1.33 (0.73,2.41)	0.352	1.38 (0.79,2.43)	0.26	1.39 (0.78,2.47)	0.264	1.35 (0.74,2.46)	0.332	38.79 (36.03,41.89)

N SNPs, number of the instrumental SNPs. The bold value indicates highlight the four MR results of insomnia and mood instability on IAs.

### Causal Effects of IAs on Psychiatric Traits

Because we could only get access to significantly associated SNPs of the summary data for anxiety disorders and mood instability, MR analyses of IAs on these two traits could not be conducted. The genetic effect sizes for the rest eight psychiatric traits vs. that on IAs for the valid IVs were displayed in Supplementary Figure S4. Although each *F* statistic was greater than 51, indicating strong instrumental effects, no causal effects of IAs on the eight psychiatric traits were found (detailed OR, 95% CI, and *p* value were shown in Table 3). Additionally, Cochran’s Q-test indicated null horizontal heterogeneity or pleiotropic effect (Supplementary Figure S4).

**TABLE 3:** The casual effects of IAs on the eight psychiatric traits by two-sample MR analyses.

Outcome	N SNPs	MR methods								*F* Statistics
		RAPS		IVW		BWMR		MR.Corr		
		*OR* (95% *CI*)	*p*	*OR* (95% *CI*)	*p*	*OR* (95% *CI*)	*p*	*OR* (95% *CI*)	*p*	
Insomnia	11	0.98 (0.96,1.00)	0.063	0.99 (0.96,1.02)	0.378	0.98 (0.96,1.00)	0.17	0.99 (0.96,1.01)	0.236	51.49 (41.44,63.40)
MDD	11	1.02 (0.98,1.06)	0.286	1.02 (0.98,1.07)	0.349	1.03 (0.98,1.07)	0.228	1.02 (0.99,1.06)	0.233	51.48 (41.41,63.38)
Subjective wellbeing	8	1.00 (0.99,1.02)	0.349	1.00 (0.98,1.02)	0.84	1.00 (0.98,1.03)	0.687	1.00 (0.99,1.02)	0.654	52.46 (40.43,67.26)
ADHD	10	0.99 (0.93,1.05)	0.75	0.99 (0.94,1.05)	0.811	0.99 (0.93,1.05)	0.806	1.00 (0.93,1.06)	0.867	53.26 (42.27,65.75)
ASD	10	1.01 (0.95,1.07)	0.836	1.01 (0.95,1.08)	0.688	1.01 (0.95,1.08)	0.709	1.01 (0.95,1.08)	0.672	53.26 (42.27,65.75)
BIP	10	1.04 (0.98,1.10)	0.217	1.04 (0.97,1.12)	0.305	1.04 (0.97,1.12)	0.288	1.04 (0.98,1.11)	0.187	53.25 (42.28,65.75)
SCZ	9	1.03 (0.99,1.08)	0.162	1.03 (0.98,1.07)	0.214	1.03 (0.98,1.08)	0.233	1.03 (0.99,1.08)	0.186	55.33 (44.01,68.64)
Neuroticism	11	0.99 (0.98,1.01)	0.325	0.99 (0.97,1.01)	0.293	0.99 (0.97,1.01)	0.356	0.99 (0.98,1.00)	0.173	51.48 (41.42,63.38)

N SNPs, number of the instrumental SNPs.

## Discussion

In this study, we firstly investigated the causal relationships between the ten psychiatric traits and IAs via two-sample bi-directional MR analyses. Our results demonstrated that insomnia had a risk effect on IAs and mood instability displayed a suggestive risk effect on IAs, whereas the other eight psychiatric traits had no significant effect on IAs. In the reverse MR analyses, no evidence implicated IAs as the cause of insomnia, MDD, subjective wellbeing, ADHD, ASD, BIP, SCZ, and neuroticism. Our instrumental variables were strong enough to avoid weak instrument bias according to the *F* statistics. The Cochran’s Q-test and scatter plots revealed null heterogeneity or pleiotropic effect.

Emerging evidence from prospective researches indicated that insomnia was correlated with increased risk of cerebral vascular disease (Wu et al., 2014; Zheng et al., 2019), including IAs. A recent meta-analysis including 23 cohorts demonstrated that insomnia significantly increased cardio-cerebral vascular events (He et al., 2017). Bakker et al. used 376 UKB phenotypes as exposure, including insomnia, to assess the risk factor of IAs in Europeans (Bakker et al., 2020). There were 20 valid SNPs used for generalized SMR (GSMR) analysis of insomnia on IAs, with the *p* = 0.0514. Here, we utilized summary data of insomnia not only from UKB but also 23andMe, expanding the valid SNPs to 159. Moreover, our four MR approaches fitted well in the linear relation between the SNPs effect on IAs and insomnia. Although inconsistent with Bakker’s result, our result indicated that insomnia had a causal risk effect on IAs. The causal relationship between insomnia and IAs reinforces the notion that prevention and early diagnosis of insomnia may help prevent IAs. However, the precise mechanism linking insomnia to IAs is unclear. Some of the proposed pathophysiological mechanisms may shed light on how insomnia might predispose an individual to IAs. Insomnia symptoms may alter cerebrovascular health through elevated circulating catecholamine (Irwin et al., 1999), sympathetic nervous activity (Dettoni et al., 2012), inflammation (Grandner M. A. et al., 2013; Grandner M. et al., 2013; Ferrie et al., 2013), endocrine or metabolic dysregulation (Okun, 2011; Westerlund et al., 2013). Clinical researches revealed that insufficient sleep might affect the levels of circulating catecholamine that influence response to emotional or physical stress (Irwin et al., 1999). Further study is needed to unravel the complex connection between insomnia and the development of IAs.

It has been reported that stroke was positively associated with anxiety (Lambiase et al., 2014), depression (Surtees et al., 2008; Henderson et al., 2013; Barlinn et al., 2015), and BIP (Prieto et al., 2014). A prospective cohort of 6,019 participants revealed higher levels of anxiety symptoms correlated with increased risk of occasional stroke (Lambiase et al., 2015). Are these psychiatric traits the risk factors of IAs (one of the main event of stroke)? However, Our results demonstrated anxiety disorders, MDD, BIP were not causally related to the development of IAs. The causal risk effect of mood instability on IAs was nominal significant according to the RAPS method (*p* = 0.047, 0.005 < *p* < 0.05). Although the *p*-values from IVW, BWMR, and MR.Corr methods were >0.05, similar effect sizes were obtained. Therefore, mood instability may be a potential risk factor for IAs. Further studies with larger sample sizes and additional mood instability genetic instruments are required to disentangle causality.

Several randomized controlled trials (RCT) reported no significant association was observed between subjective wellbeing and cardiovascular events (Lyall et al., 2018; O'Connor et al., 2020). Consistent with these observational studies, we found that subjective wellbeing had a null causal effect on IAs.

Whether there was reverse causality between psychiatric traits (depression etc.) and stroke had been debated previously (Begovac et al., 2008; Brunner et al., 2014; Lemos et al., 2020). Here, we did not detect significant genetic evidence regarding the causality of IAs to the eight psychiatric traits (insomnia, MDD, subjective wellbeing, ADHD, ASD, BIP, SCZ, and neuroticism).

More caution is needed about applying the effect size obtained by the MR method since the OR implies the mean impact of lifetime exposure. Insomnia may differ sharply across time periods. Therefore, the risk effect of insomnia on IAs is time-dependent.

Strengths of our study included the two-sample bi-directional MR analyses and the use of summary-level data from thus far the biggest sample numbers. Hence, the possible effect of reverse causality and conventional confounders could be reduced. In addition, we conducted comprehensive analyses including four MR approaches and the heterogeneity tests to avoid potential pleiotropic effects. However, it should be noted that our results were based on European populations. Thus, we should be cautious about applying this conclusion to non-Europeans since a distinct environment may significantly impact psychiatric traits and IAs.

Several limitations of the present study should also be recognized. Firstly, there was sample overlapping between the exposure and outcome because of the summary level data. Secondly, we did not conduct MR analyses of IAs on anxiety disorders and mood instability because we could only get access to significantly associated SNPs of the summary data for anxiety disorders and mood instability. Thirdly, the follow-up analysis to interpret the biological significance of the current result was lacking.

## Conclusion

In conclusion, we performed two-sample bi-directional MR analyses between psychiatric traits and IAs based on the large-scale GWAS summary statistics. Our findings provided strong evidence for a causal risk effect of insomnia on IAs and suggestive evidence for mood instability as a causal risk effect on IAs. There was no genetic support for a causal effect of the other eight psychiatric traits (anxiety disorder, MDD, subjective wellbeing, ADHD, ASD, BIP, SCZ, and neuroticism) on IAs. In the reverse MR analyses, no genetic evidence implicated IAs as the cause of insomnia, MDD, subjective wellbeing, ADHD, ASD, BIP, SCZ, and neuroticism.

## Data Availability

The original contributions presented in the study are included in the article/Supplementary Material, further inquiries can be directed to the corresponding author.
