# Supplementary material for: Investigating Causal Relationships Between Psychiatric Traits and Intracranial Aneurysms: A Bi-directional Two-Sample Mendelian Randomization Study
Source: Front Genet. 2021 Oct 19;12:741429. doi: 10.3389/fgene.2021.741429 (PMC8560679; doi:10.3389/fgene.2021.741429)
Supplement: Supplementary file 1 [file DataSheet2.docx]

| **Index of Supplements** |  |
| --- | --- |
| [**Table S1.** The definitions of the ten psychiatric traits.](#S1) | Page 3 |
| [**Table S2.** 159 valid IVs used for MR analysis of insomnia on IAs.](#S2) | Page 4 |
| [**Table S3.** 20 valid IVs used for MR analysis of mood instability on IAs.](#S3) | Page 8 |
| [**Table S4.** 5 valid IVs used for MR analysis of anxiety disorder on IAs.](#S4) | Page 9 |
| [**Table S5.** 26 valid IVs used for MR analysis of major depressive disorder on IAs.](#S5) | Page 10 |
| [**Table S6.** 32 valid IVs used for MR analysis of subjective wellbeing on IAs.](#S6) | Page 11 |
| [**Table S7.** 9 valid IVs used for MR analysis of attention deficit/hyperactivity disorder on IAs.](#S7) | Page 12 |
| [**Table S8.** 4 valid IVs used for MR analysis of autism spectrum disorder on IAs.](#S8) | Page 13 |
| [**Table S9.** 13 valid IVs used for MR analysis of bipolar disorder on IAs.](#S9) | Page 14 |
| [**Table S10.** 76 valid IVs used for MR analysis of schizophrenia on IAs.](#S10) | Page 15 |
| [**Table S11.** 19 valid IVs used for MR analysis of neuroticism on IAs.](#S11) | Page 17 |
| [**Table S12.** 11 valid IVs used for MR analysis of IAs on insomnia.](#S12) | Page 18 |
| [**Table S13.** 11 valid IVs used for MR analysis of IAs on major depressive disorder.](#S13) | Page 19 |
| [**Table S14.** 8 valid IVs used for MR analysis of IAs on subjective wellbeing.](#S14) | Page 20 |
| [**Table S15.** 10 valid IVs used for MR analysis of IAs on attention deficit/hyperactivity disorder.](#S15) | Page 21 |
| [**Table S16.** 10 valid IVs used for MR analysis of IAs on autism spectrum disorder.](#S16) | Page 22 |
| [**Table S17.** 10 valid IVs used for MR analysis of IAs on bipolar disorder.](#S17) | Page 23 |
| [**Table S18.** 9 valid IVs used for MR analysis of IAs on schizophrenia.](#S18) | Page 24 |
| [**Table S19.** 11 valid IVs used for MR analysis of IAs on neuroticism.](#S19) | Page 25 |

**Table S1. The definitions of the ten psychiatric traits.**

| **Trait** | **GWAS Phenotype Definition** |
| --- | --- |
| Insomnia | Sample whose answer is “usually” to question “Do you have trouble falling asleep at night or do you wake up in the middle of the night?” will be included as a case. |
| Mood instability | Sample whose answer is “yes” to question “Does your mood often go up and down?” will be included as a case. |
| Anxiety disorders | Cases met one of two definitions. First was self-reporting a lifetime professional diagnosis of an anxiety disorder. Second was meeting criteria for a likely lifetime diagnosis of DSM-IV generalised anxiety disorder. |
| MDD | Cases were required to meet international consensus criteria (DSM-IV, ICD-9, or ICD-10) for a lifetime diagnosis of MDD established using structured diagnostic instruments from assessments by trained interviewers, clinician administered checklists, or medical record review. |
| Subjective wellbeing | A question of “How satisfied are you with your life?” with five options to choose (“Very dissatisfied,” “Somewhat dissatisfied,” “Neither dissatisfied nor satisfied,” “Somewhat satisfied,” “Very satisfied”). |
| ADHD | Cases were diagnosed by psychiatrists at in- or out-patient clinics, predominantly the latter according to ICD10 (F90.0 diagnosis code). |
| ASD | For PGC sample, cases were those met from either the Autism Diagnostic Interview-Revised (ADI-R) or the Autism Diagnostic Observation Schedule (ADOS) domain scores. |
| BIP | Cases were required to meet international consensus criteria (DSM-IV, ICD-9, or ICD-10) for a lifetime diagnosis of BIP established using structured diagnostic instruments from assessments by trained interviewers, clinician-administered checklists, or medical record review. |
| SCZ | Cases validation acquired both clinical and consensus research diagnosis. Details of cases validation were given in supplementary materials (PMID: 29483656). |
| Neuroticism | A scale with 12 items measures the degree of neuroticism. |

MDD major depressive disorder, ADHD attention deficit/hyperactivity disorder, ASD autism spectrum disorder, BIP bipolar disorder, SCZ schizophrenia.

**Table S2. 159 valid IVs used for MR analysis of insomnia on IAs.**

| SNP | Effect allele | Non-effect allele | Effect allele frequency | Beta | SE | *P* |
| --- | --- | --- | --- | --- | --- | --- |
| rs1015438 | A | G | 0.1882 | 0.058269 | 0.008 | 2.51E-14 |
| rs1031654 | C | A | 0.2004 | 0.051293 | 0.007 | 3.88E-12 |
| rs1038093 | T | C | 0.6282 | 0.039221 | 0.006 | 2.47E-10 |
| rs10502966 | G | A | 0.418 | 0.038741 | 0.006 | 8.54E-11 |
| rs1064213 | G | A | 0.5211 | 0.036664 | 0.006 | 6.41E-10 |
| rs10756571 | T | C | 0.6853 | 0.036332 | 0.006 | 1.80E-08 |
| rs10758593 | G | A | 0.6011 | 0.035627 | 0.006 | 4.90E-09 |
| rs10761240 | G | A | 0.6037 | 0.042908 | 0.006 | 2.12E-12 |
| rs10800992 | T | C | 0.4431 | 0.042101 | 0.006 | 3.84E-12 |
| rs10825503 | T | G | 0.4873 | 0.033435 | 0.006 | 1.43E-08 |
| rs10865954 | T | C | 0.3344 | 0.042101 | 0.006 | 1.92E-11 |
| rs10928256 | T | C | 0.4192 | 0.034401 | 0.006 | 1.61E-08 |
| rs10944696 | G | A | 0.7022 | 0.037702 | 0.007 | 7.99E-09 |
| rs10947987 | C | T | 0.5569 | 0.032523 | 0.006 | 4.08E-08 |
| rs10955647 | T | G | 0.5321 | 0.033435 | 0.006 | 1.84E-08 |
| rs11090039 | A | G | 0.2871 | 0.039221 | 0.007 | 1.82E-09 |
| rs11119409 | C | T | 0.4134 | 0.034591 | 0.006 | 1.19E-08 |
| rs11149313 | A | G | 0.7297 | 0.040182 | 0.007 | 2.38E-09 |
| rs1147852 | A | G | 0.3095 | 0.039221 | 0.006 | 9.94E-10 |
| rs11588755 | G | A | 0.478 | 0.034591 | 0.006 | 5.14E-09 |
| rs11605348 | G | A | 0.6505 | 0.044997 | 0.006 | 7.01E-13 |
| rs116466468 | T | C | 0.7593 | 0.044017 | 0.007 | 2.11E-10 |
| rs1167132 | T | C | 0.3917 | 0.035367 | 0.006 | 8.73E-09 |
| rs11679943 | A | G | 0.3471 | 0.037296 | 0.006 | 3.16E-09 |
| rs11722569 | T | C | 0.6586 | 0.034401 | 0.006 | 2.91E-08 |
| rs11803128 | G | A | 0.3459 | 0.040822 | 0.006 | 6.85E-11 |
| rs118166957 | T | C | 0.1591 | 0.067659 | 0.008 | 1.95E-16 |
| rs11838830 | G | A | 0.0564 | 0.080126 | 0.013 | 5.20E-10 |
| rs12187443 | T | C | 0.6681 | 0.040182 | 0.006 | 1.64E-10 |
| rs12310246 | A | G | 0.2489 | 0.044973 | 0.007 | 4.74E-11 |
| rs12520974 | C | T | 0.5154 | 0.035627 | 0.006 | 1.69E-09 |
| rs12605642 | T | G | 0.4864 | 0.035367 | 0.006 | 2.13E-09 |
| rs12666306 | A | G | 0.5017 | 0.042101 | 0.006 | 2.24E-12 |
| rs12790660 | C | T | 0.3156 | 0.039781 | 0.006 | 4.49E-10 |
| rs1289939 | C | T | 0.7675 | 0.040822 | 0.007 | 6.00E-09 |
| rs12912299 | C | T | 0.5107 | 0.042908 | 0.006 | 4.42E-13 |
| rs12917449 | C | A | 0.1939 | 0.041864 | 0.008 | 2.97E-08 |
| rs12924275 | T | C | 0.268 | 0.038259 | 0.007 | 1.93E-08 |
| rs12983032 | G | A | 0.6566 | 0.042908 | 0.006 | 1.07E-11 |
| rs13010288 | G | T | 0.8674 | 0.05975 | 0.009 | 9.26E-12 |
| rs13135092 | G | A | 0.0825 | 0.088831 | 0.011 | 2.53E-16 |
| rs13138995 | A | G | 0.3896 | 0.034401 | 0.006 | 1.97E-08 |
| rs1357685 | T | C | 0.4735 | 0.033435 | 0.006 | 1.39E-08 |
| rs1530938 | A | G | 0.4423 | 0.036332 | 0.006 | 8.82E-10 |
| rs1536053 | C | T | 0.6843 | 0.037702 | 0.006 | 6.04E-09 |
| rs1553754 | G | T | 0.4379 | 0.033557 | 0.006 | 3.51E-08 |
| rs1567084 | A | G | 0.4981 | 0.033435 | 0.006 | 2.14E-08 |
| rs1580173 | A | G | 0.5608 | 0.033435 | 0.006 | 2.28E-08 |
| rs1620977 | A | G | 0.2696 | 0.051643 | 0.007 | 2.27E-14 |
| rs16903122 | T | C | 0.2487 | 0.055435 | 0.007 | 9.04E-16 |
| rs17005118 | A | G | 0.264 | 0.042101 | 0.007 | 6.13E-10 |
| rs17025198 | A | G | 0.2043 | 0.041142 | 0.007 | 2.19E-08 |
| rs17223714 | A | G | 0.7885 | 0.045929 | 0.007 | 2.44E-10 |
| rs17367725 | C | T | 0.6487 | 0.035627 | 0.006 | 9.29E-09 |
| rs176644 | T | G | 0.4036 | 0.035367 | 0.006 | 9.49E-09 |
| rs1861412 | A | G | 0.4338 | 0.038259 | 0.006 | 1.67E-10 |
| rs190073 | G | A | 0.5859 | 0.033557 | 0.006 | 2.86E-08 |
| rs1927902 | T | C | 0.2542 | 0.052592 | 0.007 | 1.15E-14 |
| rs224029 | C | T | 0.6005 | 0.038741 | 0.006 | 2.51E-10 |
| rs2286729 | A | G | 0.0862 | 0.069526 | 0.011 | 5.37E-11 |
| rs2364921 | C | T | 0.5309 | 0.033557 | 0.006 | 2.13E-08 |
| rs2388840 | G | A | 0.4243 | 0.036664 | 0.006 | 1.37E-09 |
| rs2389631 | C | A | 0.3334 | 0.039781 | 0.006 | 2.03E-10 |
| rs2431108 | C | T | 0.328 | 0.053401 | 0.006 | 7.83E-17 |
| rs2447094 | C | A | 0.5313 | 0.033557 | 0.006 | 2.50E-08 |
| rs2491124 | T | C | 0.5758 | 0.04879 | 0.006 | 8.81E-16 |
| rs2598293 | T | C | 0.4763 | 0.035367 | 0.006 | 2.48E-09 |
| rs2737240 | A | G | 0.7076 | 0.036332 | 0.007 | 3.37E-08 |
| rs2815757 | T | C | 0.8089 | 0.055435 | 0.008 | 2.24E-13 |
| rs2838787 | G | A | 0.6076 | 0.035627 | 0.006 | 7.65E-09 |
| rs28552587 | A | G | 0.5641 | 0.033435 | 0.006 | 3.30E-08 |
| rs28582096 | G | A | 0.795 | 0.054456 | 0.007 | 1.74E-13 |
| rs2903385 | A | G | 0.4844 | 0.043059 | 0.006 | 4.53E-13 |
| rs314281 | C | T | 0.5469 | 0.042908 | 0.006 | 6.03E-13 |
| rs3184470 | G | A | 0.6493 | 0.037702 | 0.006 | 9.73E-10 |
| rs324017 | A | C | 0.294 | 0.039221 | 0.007 | 1.61E-09 |
| rs34036083 | C | T | 0.3424 | 0.035627 | 0.006 | 2.07E-08 |
| rs34214423 | A | C | 0.8087 | 0.044973 | 0.008 | 3.18E-09 |
| rs34967082 | A | G | 0.4136 | 0.035367 | 0.006 | 4.34E-09 |
| rs35110063 | A | G | 0.4266 | 0.039221 | 0.006 | 8.82E-11 |
| rs35322724 | A | C | 0.5774 | 0.04879 | 0.006 | 3.75E-16 |
| rs35539975 | A | G | 0.7785 | 0.042101 | 0.007 | 4.49E-09 |
| rs3774751 | G | T | 0.5379 | 0.040822 | 0.006 | 7.32E-12 |
| rs3902952 | T | C | 0.1881 | 0.047837 | 0.008 | 2.55E-10 |
| rs4090240 | C | T | 0.7225 | 0.038741 | 0.007 | 8.46E-09 |
| rs4238755 | C | A | 0.7362 | 0.042908 | 0.007 | 2.30E-10 |
| rs4260410 | T | C | 0.332 | 0.034401 | 0.006 | 4.87E-08 |
| rs429358 | T | C | 0.8458 | 0.045929 | 0.008 | 2.13E-08 |
| rs4502882 | C | T | 0.3422 | 0.038741 | 0.006 | 7.96E-10 |
| rs4588900 | A | G | 0.5164 | 0.033435 | 0.006 | 1.57E-08 |
| rs4592425 | T | G | 0.6965 | 0.040182 | 0.006 | 4.31E-10 |
| rs4643373 | T | C | 0.7006 | 0.041142 | 0.007 | 1.58E-10 |
| rs4664299 | C | T | 0.7651 | 0.040822 | 0.007 | 4.95E-09 |
| rs4702 | G | A | 0.4438 | 0.04814 | 0.006 | 6.78E-16 |
| rs4709655 | C | T | 0.8809 | 0.054456 | 0.009 | 3.09E-09 |
| rs4767645 | G | T | 0.5386 | 0.036664 | 0.006 | 6.47E-10 |
| rs492858 | C | T | 0.9241 | 0.06614 | 0.011 | 3.46E-09 |
| rs521484 | G | A | 0.2332 | 0.039781 | 0.007 | 1.53E-08 |
| rs524859 | G | A | 0.6399 | 0.043952 | 0.006 | 1.48E-12 |
| rs55772859 | A | C | 0.3106 | 0.042101 | 0.006 | 4.82E-11 |
| rs55972276 | A | C | 0.1366 | 0.07325 | 0.009 | 4.19E-17 |
| rs56097173 | T | C | 0.6808 | 0.040182 | 0.006 | 2.69E-10 |
| rs56133505 | A | G | 0.5369 | 0.041142 | 0.006 | 5.59E-12 |
| rs566673 | G | T | 0.4649 | 0.038741 | 0.006 | 1.18E-10 |
| rs5877 | T | C | 0.6691 | 0.036332 | 0.006 | 1.23E-08 |
| rs6019663 | T | C | 0.293 | 0.040182 | 0.007 | 6.47E-10 |
| rs60565673 | G | T | 0.3789 | 0.042908 | 0.006 | 1.59E-12 |
| rs61921611 | C | T | 0.308 | 0.043952 | 0.006 | 7.84E-12 |
| rs62158170 | A | G | 0.7856 | 0.065788 | 0.007 | 1.20E-19 |
| rs62264767 | A | C | 0.8531 | 0.064851 | 0.008 | 1.63E-14 |
| rs623025 | C | T | 0.7448 | 0.037702 | 0.007 | 3.16E-08 |
| rs62429521 | A | C | 0.1456 | 0.050693 | 0.008 | 1.78E-09 |
| rs6465151 | T | C | 0.1134 | 0.05638 | 0.009 | 1.90E-09 |
| rs647905 | T | C | 0.5409 | 0.033435 | 0.006 | 2.87E-08 |
| rs6510033 | G | A | 0.2747 | 0.036664 | 0.007 | 4.66E-08 |
| rs6562066 | T | C | 0.3688 | 0.039221 | 0.006 | 1.38E-10 |
| rs6589988 | G | A | 0.3243 | 0.037702 | 0.006 | 4.70E-09 |
| rs6597649 | T | C | 0.3994 | 0.033435 | 0.006 | 3.05E-08 |
| rs6601080 | A | G | 0.6762 | 0.035367 | 0.006 | 2.21E-08 |
| rs667730 | T | C | 0.5788 | 0.033435 | 0.006 | 2.26E-08 |
| rs6702604 | G | A | 0.4157 | 0.036664 | 0.006 | 1.30E-09 |
| rs671985 | G | A | 0.5484 | 0.037702 | 0.006 | 2.79E-10 |
| rs6808140 | T | C | 0.5053 | 0.039221 | 0.006 | 5.35E-11 |
| rs694786 | C | T | 0.5395 | 0.043952 | 0.006 | 1.97E-13 |
| rs6967168 | G | T | 0.2456 | 0.043952 | 0.007 | 1.39E-10 |
| rs6978112 | T | C | 0.4105 | 0.034401 | 0.006 | 2.11E-08 |
| rs699844 | A | G | 0.9195 | 0.060154 | 0.011 | 4.11E-08 |
| rs701394 | G | A | 0.3624 | 0.035627 | 0.006 | 6.83E-09 |
| rs7040224 | A | G | 0.3161 | 0.037296 | 0.006 | 4.24E-09 |
| rs715338 | A | G | 0.5776 | 0.041142 | 0.006 | 7.85E-12 |
| rs728017 | G | A | 0.6136 | 0.034591 | 0.006 | 9.51E-09 |
| rs72820274 | A | G | 0.417 | 0.034401 | 0.006 | 1.28E-08 |
| rs72899452 | T | C | 0.0648 | 0.074179 | 0.012 | 1.00E-09 |
| rs73079014 | C | T | 0.874 | 0.04919 | 0.009 | 3.65E-08 |
| rs73163783 | C | T | 0.2768 | 0.037702 | 0.007 | 1.39E-08 |
| rs73671843 | G | A | 0.8743 | 0.055513 | 0.009 | 5.49E-10 |
| rs7402939 | C | T | 0.624 | 0.035627 | 0.006 | 5.19E-09 |
| rs7432782 | C | T | 0.0442 | 0.083382 | 0.014 | 7.42E-09 |
| rs7486418 | T | G | 0.657 | 0.041142 | 0.006 | 6.84E-11 |
| rs75452188 | A | G | 0.878 | 0.051643 | 0.009 | 1.58E-08 |
| rs7566062 | T | C | 0.2248 | 0.059212 | 0.007 | 1.37E-16 |
| rs7571486 | G | A | 0.749 | 0.038741 | 0.007 | 1.40E-08 |
| rs75932578 | C | T | 0.7841 | 0.039781 | 0.007 | 4.15E-08 |
| rs7599697 | C | T | 0.6417 | 0.036664 | 0.006 | 5.00E-09 |
| rs7625896 | A | G | 0.6545 | 0.036332 | 0.006 | 5.28E-09 |
| rs78206187 | G | A | 0.0558 | 0.094311 | 0.013 | 2.96E-13 |
| rs8076183 | C | T | 0.5515 | 0.037702 | 0.006 | 2.75E-10 |
| rs8181889 | G | A | 0.6008 | 0.037702 | 0.006 | 8.90E-10 |
| rs823247 | C | T | 0.521 | 0.036664 | 0.006 | 5.25E-10 |
| rs871994 | A | C | 0.4352 | 0.035367 | 0.006 | 5.50E-09 |
| rs874168 | T | C | 0.5254 | 0.034401 | 0.006 | 7.95E-09 |
| rs908668 | T | C | 0.2081 | 0.049742 | 0.007 | 1.41E-11 |
| rs910187 | G | A | 0.627 | 0.034591 | 0.006 | 1.63E-08 |
| rs940780 | T | C | 0.3588 | 0.038259 | 0.006 | 8.50E-10 |
| rs9527083 | G | A | 0.3295 | 0.075802 | 0.006 | 1.61E-32 |
| rs9563886 | C | T | 0.3937 | 0.033557 | 0.006 | 3.08E-08 |
| rs9889282 | C | A | 0.3871 | 0.041864 | 0.006 | 4.70E-12 |
| rs9931543 | T | C | 0.7359 | 0.047837 | 0.007 | 1.11E-12 |
| rs9964420 | A | C | 0.301 | 0.035367 | 0.007 | 4.54E-08 |

**Table S3. 20 valid IVs used for MR analysis of mood instability on IAs.**

| SNP | Effect allele | Non-effect allele | Effect allele frequency | Beta | SE | *P* |
| --- | --- | --- | --- | --- | --- | --- |
| rs1050863 | G | A | 0.5 | 0.008127 | 0.001161 | 2.60E-12 |
| rs11599236 | T | C | 0.5 | 0.007295 | 0.001175 | 5.40E-10 |
| rs12477961 | A | G | 0.5 | 0.006424 | 0.001163 | 3.30E-08 |
| rs12486699 | A | C | 0.5 | 0.007323 | 0.001342 | 4.80E-08 |
| rs1559422 | C | T | 0.5 | 0.006862 | 0.001241 | 3.20E-08 |
| rs16932966 | A | G | 0.5 | 0.008295 | 0.001318 | 3.10E-10 |
| rs1962104 | C | T | 0.5 | 0.007951 | 0.001162 | 7.70E-12 |
| rs2483509 | A | G | 0.5 | 0.006462 | 0.001151 | 2.00E-08 |
| rs28655666 | G | A | 0.5 | 0.006918 | 0.001149 | 1.70E-09 |
| rs4309187 | C | A | 0.5 | 0.00929 | 0.001236 | 5.70E-14 |
| rs45510500 | C | T | 0.5 | 0.013758 | 0.002491 | 3.30E-08 |
| rs4799949 | C | T | 0.5 | 0.007521 | 0.001217 | 6.50E-10 |
| rs56116032 | A | G | 0.5 | 0.009023 | 0.001379 | 6.10E-11 |
| rs56403421 | C | A | 0.5 | 0.008123 | 0.001223 | 3.10E-11 |
| rs613872 | T | G | 0.5 | 0.011684 | 0.001509 | 9.90E-15 |
| rs61915924 | A | C | 0.5 | 0.007548 | 0.001329 | 1.30E-08 |
| rs67447472 | T | G | 0.5 | 0.011417 | 0.001938 | 3.80E-09 |
| rs6861117 | A | G | 0.5 | 0.012247 | 0.002175 | 1.80E-08 |
| rs73082357 | G | A | 0.5 | 0.011724 | 0.001777 | 4.20E-11 |
| rs8045174 | T | C | 0.5 | 0.008513 | 0.001394 | 1.00E-09 |

**Table S4. 5 valid IVs used for MR analysis of anxiety disorder on IAs.**

| SNP | Effect allele | Non-effect allele | Effect allele frequency | Beta | SE | *P* |
| --- | --- | --- | --- | --- | --- | --- |
| rs10959883 | T | C | 0.91 | 0.15 | 0.022551 | 2.90E-11 |
| rs1187280 | G | A | 0.88 | 0.13 | 0.022411 | 6.60E-09 |
| rs2861139 | C | T | 0.62 | 0.13 | 0.02183 | 2.60E-09 |
| rs3807866 | A | G | 0.48 | 0.12 | 0.021984 | 4.80E-08 |
| rs4855559 | G | T | 0.79 | 0.12 | 0.0218 | 3.70E-08 |

**Table S5. 26 valid IVs used for MR analysis of major depressive disorder on IAs.**

| SNP | Effect allele | Non-effect allele | Effect allele frequency | Beta | SE | *P* |
| --- | --- | --- | --- | --- | --- | --- |
| rs11135349 | C | A | 0.52 | 0.030459 | 0.0048 | 1.10E-09 |
| rs11643192 | A | C | 0.41 | 0.029559 | 0.0049 | 3.40E-08 |
| rs11663393 | A | G | 0.45 | 0.029559 | 0.0049 | 1.60E-08 |
| rs11682175 | C | T | 0.48 | 0.030459 | 0.0048 | 4.70E-09 |
| rs1226412 | T | C | 0.79 | 0.029559 | 0.0059 | 2.40E-08 |
| rs12552 | A | G | 0.44 | 0.039221 | 0.0048 | 6.10E-19 |
| rs12666117 | A | G | 0.47 | 0.029559 | 0.0048 | 1.40E-08 |
| rs12958048 | A | G | 0.33 | 0.029559 | 0.0051 | 3.60E-11 |
| rs1354115 | A | C | 0.62 | 0.029559 | 0.0049 | 2.40E-08 |
| rs1432639 | A | C | 0.63 | 0.039221 | 0.005 | 4.60E-15 |
| rs159963 | C | A | 0.44 | 0.030459 | 0.0049 | 3.20E-08 |
| rs1806153 | T | G | 0.22 | 0.039221 | 0.0059 | 1.20E-09 |
| rs2389016 | T | C | 0.28 | 0.029559 | 0.0053 | 1.00E-08 |
| rs4074723 | C | A | 0.59 | 0.030459 | 0.0049 | 3.10E-08 |
| rs4261101 | G | A | 0.63 | 0.030459 | 0.005 | 1.00E-08 |
| rs4869056 | G | A | 0.37 | 0.030459 | 0.005 | 6.80E-09 |
| rs4904738 | C | T | 0.43 | 0.030459 | 0.0049 | 2.60E-09 |
| rs5758265 | A | G | 0.28 | 0.029559 | 0.0054 | 7.60E-09 |
| rs61867293 | C | T | 0.8 | 0.040822 | 0.0061 | 7.00E-10 |
| rs7029033 | T | C | 0.07 | 0.04879 | 0.0093 | 2.70E-08 |
| rs7198928 | T | C | 0.62 | 0.029559 | 0.005 | 1.00E-08 |
| rs7200826 | T | C | 0.25 | 0.029559 | 0.0055 | 2.40E-08 |
| rs7430565 | G | A | 0.42 | 0.030459 | 0.0048 | 2.90E-09 |
| rs7856424 | C | T | 0.71 | 0.030459 | 0.0053 | 8.50E-09 |
| rs8025231 | C | A | 0.43 | 0.030459 | 0.0048 | 2.40E-12 |
| rs915057 | G | A | 0.58 | 0.030459 | 0.0049 | 7.60E-10 |

**Table S6. 32 valid IVs used for MR analysis of subjective wellbeing on IAs.**

| SNP | Effect allele | Non-effect allele | Effect allele frequency | Beta | SE | *P* |
| --- | --- | --- | --- | --- | --- | --- |
| rs10434704 | T | C | 0.4711 | 0.0101 | 0.0018 | 4.72E-08 |
| rs10498870 | A | G | 0.299 | 0.011 | 0.002 | 4.13E-08 |
| rs10514301 | C | T | 0.8757 | 0.0181 | 0.0028 | 9.64E-11 |
| rs10920678 | G | A | 0.5721 | 0.0125 | 0.0019 | 2.04E-11 |
| rs10953620 | A | C | 0.5218 | 0.0101 | 0.0018 | 4.81E-08 |
| rs11665070 | A | G | 0.6693 | 0.0116 | 0.002 | 3.23E-09 |
| rs11855468 | A | G | 0.952 | 0.0247 | 0.0043 | 1.20E-08 |
| rs12187898 | T | C | 0.5191 | 0.0129 | 0.0018 | 2.34E-12 |
| rs12642606 | G | A | 0.5038 | 0.0121 | 0.0018 | 5.70E-11 |
| rs1400867 | A | G | 0.1267 | 0.0168 | 0.0028 | 1.26E-09 |
| rs172404 | A | C | 0.6073 | 0.0113 | 0.0019 | 1.99E-09 |
| rs1941687 | A | C | 0.5982 | 0.0117 | 0.0019 | 4.74E-10 |
| rs1961639 | A | G | 0.6631 | 0.0137 | 0.0019 | 2.26E-12 |
| rs2075679 | C | T | 0.4257 | 0.0117 | 0.0019 | 4.16E-10 |
| rs2243616 | T | G | 0.6464 | 0.0107 | 0.002 | 4.47E-08 |
| rs35995292 | T | G | 0.6809 | 0.011 | 0.002 | 2.83E-08 |
| rs3824535 | G | T | 0.1257 | 0.0165 | 0.0028 | 4.16E-09 |
| rs4442212 | C | T | 0.5601 | 0.0108 | 0.0019 | 6.85E-09 |
| rs4478137 | A | G | 0.6298 | 0.012 | 0.0019 | 2.90E-10 |
| rs531639 | T | C | 0.6282 | 0.0113 | 0.0019 | 6.56E-09 |
| rs5877 | C | T | 0.34 | 0.011 | 0.002 | 1.84E-08 |
| rs6063085 | C | A | 0.3689 | 0.0125 | 0.0019 | 6.05E-11 |
| rs6579956 | G | T | 0.525 | 0.0118 | 0.0018 | 1.43E-10 |
| rs6759922 | G | A | 0.5494 | 0.0104 | 0.0019 | 2.66E-08 |
| rs677325 | C | A | 0.8681 | 0.0154 | 0.0027 | 1.46E-08 |
| rs6787782 | G | T | 0.859 | 0.0149 | 0.0026 | 2.00E-08 |
| rs703410 | A | G | 0.2625 | 0.0118 | 0.0021 | 2.20E-08 |
| rs7111031 | C | A | 0.363 | 0.0115 | 0.0019 | 2.67E-09 |
| rs7617480 | C | A | 0.7706 | 0.0128 | 0.0022 | 8.51E-09 |
| rs8084351 | G | A | 0.4822 | 0.013 | 0.0019 | 3.54E-12 |
| rs815753 | T | C | 0.6978 | 0.0119 | 0.002 | 4.20E-09 |
| rs993884 | T | C | 0.6283 | 0.0114 | 0.0019 | 2.89E-09 |

**Table S7. 9 valid IVs used for MR analysis of attention deficit/hyperactivity disorder on IAs.**

| SNP | Effect allele | Non-effect allele | Effect allele frequency | Beta | SE | *P* |
| --- | --- | --- | --- | --- | --- | --- |
| rs10262192 | A | G | 0.432 | 0.074096 | 0.0135 | 3.66E-08 |
| rs112361411 | C | T | 0.448 | 0.105306 | 0.0148 | 1.13E-12 |
| rs1222064 | T | C | 0.2685 | 0.076804 | 0.0155 | 6.85E-07 |
| rs1427829 | A | G | 0.4607 | 0.082197 | 0.0136 | 1.35E-09 |
| rs16884473 | C | T | 0.5 | 0.068098 | 0.0138 | 8.56E-07 |
| rs4858241 | T | G | 0.38 | 0.082197 | 0.0143 | 8.17E-09 |
| rs4916723 | C | A | 0.338 | 0.077803 | 0.0138 | 1.81E-08 |
| rs74760947 | G | A | 0.026 | 0.17961 | 0.0317 | 1.39E-08 |
| rs9677504 | A | G | 0.115 | 0.113498 | 0.0213 | 9.83E-08 |

**Table S8. 4 valid IVs used for MR analysis of autism spectrum disorder on IAs.**

| SNP | Effect allele | Non-effect allele | Effect allele frequency | Beta | SE | *P* |
| --- | --- | --- | --- | --- | --- | --- |
| rs1452075 | T | C | 0.721 | 0.061 | 0.01 | 3.17E-09 |
| rs1620977 | A | G | 0.26 | 0.056 | 0.01 | 6.66E-09 |
| rs16854048 | A | C | 0.858 | 0.069 | 0.012 | 1.29E-08 |
| rs2388334 | G | A | 0.483 | 0.065 | 0.009 | 3.34E-12 |

**Table S9. 13 valid IVs used for MR analysis of bipolar disorder on IAs.**

| SNP | Effect allele | Non-effect allele | Effect allele frequency | Beta | SE | *P* |
| --- | --- | --- | --- | --- | --- | --- |
| rs10035291 | T | C | 0.675 | 0.067799 | 0.0122 | 2.67E-08 |
| rs10744560 | T | C | 0.336 | 0.073204 | 0.0117 | 3.62E-10 |
| rs10896090 | A | G | 0.809 | 0.0804 | 0.0143 | 1.90E-08 |
| rs113779084 | A | G | 0.304 | 0.072897 | 0.0122 | 2.51E-09 |
| rs11557713 | A | G | 0.285 | 0.067098 | 0.0122 | 3.64E-08 |
| rs11647445 | G | T | 0.346 | 0.075597 | 0.0117 | 1.08E-10 |
| rs11724116 | C | T | 0.84 | 0.085002 | 0.0152 | 2.37E-08 |
| rs12575685 | A | G | 0.314 | 0.070197 | 0.0122 | 7.71E-09 |
| rs17183814 | G | A | 0.9251 | 0.132503 | 0.0221 | 2.02E-09 |
| rs2388334 | G | A | 0.481 | 0.064997 | 0.0111 | 4.01E-09 |
| rs3804640 | A | G | 0.533 | 0.062796 | 0.0112 | 1.99E-08 |
| rs7122539 | G | A | 0.652 | 0.064603 | 0.0118 | 3.77E-08 |
| rs9834970 | C | T | 0.491 | 0.077302 | 0.0112 | 5.72E-12 |

**TableS 10. 76 valid IVs used for MR analysis of schizophrenia on IAs.**

| SNP | Effect allele | Non-effect allele | Effect allele frequency | Beta | SE | *P* |
| --- | --- | --- | --- | --- | --- | --- |
| rs10148671 | C | T | 0.5 | 0.06294 | 0.01 | 5.46E-10 |
| rs1042992 | T | C | 0.5 | 0.073647 | 0.012 | 3.67E-09 |
| rs10783624 | C | A | 0.5 | 0.060154 | 0.01 | 5.44E-09 |
| rs1080500 | G | A | 0.5 | 0.072321 | 0.01 | 2.71E-12 |
| rs10985817 | C | T | 0.5 | 0.080126 | 0.013 | 1.02E-09 |
| rs111294930 | A | G | 0.5 | 0.082501 | 0.012 | 9.04E-12 |
| rs11165867 | T | C | 0.5 | 0.06935 | 0.013 | 3.87E-08 |
| rs11783093 | C | T | 0.5 | 0.09349 | 0.014 | 7.64E-12 |
| rs12129719 | A | G | 0.5 | 0.054456 | 0.01 | 3.35E-08 |
| rs12293670 | A | G | 0.5 | 0.080658 | 0.01 | 1.70E-15 |
| rs12447542 | A | G | 0.5 | 0.085558 | 0.015 | 1.44E-08 |
| rs12704290 | G | A | 0.5 | 0.114221 | 0.015 | 3.57E-14 |
| rs12712510 | T | C | 0.5 | 0.057325 | 0.01 | 8.18E-09 |
| rs12898315 | A | G | 0.5 | 0.05657 | 0.01 | 2.51E-09 |
| rs12908161 | A | G | 0.5 | 0.066724 | 0.011 | 9.41E-10 |
| rs12991836 | C | A | 0.5 | 0.060812 | 0.01 | 6.46E-10 |
| rs13107325 | T | C | 0.5 | 0.160169 | 0.019 | 1.19E-16 |
| rs13121251 | T | C | 0.5 | 0.057325 | 0.01 | 4.06E-08 |
| rs13169274 | C | T | 0.5 | 0.05975 | 0.01 | 7.06E-10 |
| rs1319017 | A | G | 0.5 | 0.06614 | 0.01 | 7.82E-11 |
| rs140505938 | C | T | 0.5 | 0.083422 | 0.014 | 6.50E-10 |
| rs14403 | C | T | 0.5 | 0.074179 | 0.012 | 1.71E-10 |
| rs1473594 | T | C | 0.5 | 0.063913 | 0.01 | 3.33E-11 |
| rs16867576 | A | G | 0.5 | 0.10075 | 0.015 | 1.65E-11 |
| rs17465671 | C | A | 0.5 | 0.057325 | 0.01 | 4.14E-09 |
| rs17514846 | C | A | 0.5 | 0.068279 | 0.01 | 2.55E-12 |
| rs1765142 | A | C | 0.5 | 0.057629 | 0.01 | 1.13E-08 |
| rs1975802 | G | A | 0.5 | 0.06935 | 0.013 | 3.56E-08 |
| rs2007044 | G | A | 0.5 | 0.088831 | 0.01 | 5.63E-20 |
| rs2053079 | G | A | 0.5 | 0.070422 | 0.011 | 1.82E-10 |
| rs211829 | T | C | 0.5 | 0.059212 | 0.01 | 2.29E-09 |
| rs2161711 | A | G | 0.5 | 0.069526 | 0.013 | 4.22E-08 |
| rs217287 | C | T | 0.5 | 0.069526 | 0.01 | 9.53E-13 |
| rs2410572 | G | A | 0.5 | 0.054488 | 0.01 | 1.07E-08 |
| rs2514218 | A | G | 0.5 | 0.086648 | 0.012 | 2.42E-12 |
| rs2917569 | T | C | 0.5 | 0.061095 | 0.01 | 3.11E-10 |
| rs2949006 | T | G | 0.5 | 0.099845 | 0.012 | 3.69E-17 |
| rs2970610 | T | C | 0.5 | 0.067659 | 0.01 | 1.39E-11 |
| rs312477 | G | A | 0.5 | 0.064851 | 0.011 | 1.38E-08 |
| rs34796896 | G | A | 0.5 | 0.083422 | 0.012 | 3.19E-12 |
| rs35346733 | C | T | 0.5 | 0.072321 | 0.01 | 2.42E-12 |
| rs36043959 | G | A | 0.5 | 0.067659 | 0.01 | 4.07E-12 |
| rs3735025 | T | C | 0.5 | 0.064851 | 0.01 | 7.02E-11 |
| rs489939 | G | A | 0.5 | 0.057325 | 0.01 | 1.24E-08 |
| rs4925114 | A | G | 0.5 | 0.05638 | 0.01 | 2.64E-08 |
| rs4936215 | A | G | 0.5 | 0.091667 | 0.012 | 5.32E-14 |
| rs55669358 | C | T | 0.5 | 0.09541 | 0.017 | 1.37E-08 |
| rs56282503 | C | T | 0.5 | 0.061875 | 0.011 | 2.30E-08 |
| rs56775891 | T | C | 0.5 | 0.06614 | 0.011 | 2.03E-09 |
| rs5757730 | G | A | 0.5 | 0.071496 | 0.01 | 1.76E-12 |
| rs58950470 | T | G | 0.5 | 0.057629 | 0.01 | 2.07E-08 |
| rs6002655 | T | C | 0.5 | 0.074724 | 0.01 | 2.15E-14 |
| rs6035706 | G | A | 0.5 | 0.05975 | 0.01 | 7.24E-09 |
| rs6065094 | G | A | 0.5 | 0.085558 | 0.01 | 7.91E-17 |
| rs62334820 | T | C | 0.5 | 0.08121 | 0.012 | 9.60E-12 |
| rs634940 | T | G | 0.5 | 0.06294 | 0.011 | 1.30E-08 |
| rs6434928 | G | A | 0.5 | 0.07325 | 0.01 | 3.62E-13 |
| rs6680011 | C | A | 0.5 | 0.073647 | 0.013 | 2.83E-08 |
| rs6800435 | A | C | 0.5 | 0.082295 | 0.015 | 2.00E-08 |
| rs704373 | A | G | 0.5 | 0.064851 | 0.01 | 1.39E-10 |
| rs7191183 | C | T | 0.5 | 0.05975 | 0.01 | 6.31E-09 |
| rs7225476 | A | G | 0.5 | 0.052346 | 0.01 | 4.86E-08 |
| rs72769124 | A | C | 0.5 | 0.108699 | 0.017 | 4.73E-10 |
| rs7432375 | A | G | 0.5 | 0.080126 | 0.012 | 4.07E-12 |
| rs7499750 | A | C | 0.5 | 0.070458 | 0.011 | 4.24E-10 |
| rs7508148 | T | C | 0.5 | 0.076961 | 0.012 | 4.06E-11 |
| rs7596038 | C | T | 0.5 | 0.067659 | 0.01 | 2.37E-12 |
| rs75968099 | T | C | 0.5 | 0.06614 | 0.01 | 9.41E-11 |
| rs760608 | G | A | 0.5 | 0.061095 | 0.011 | 1.90E-08 |
| rs7632921 | G | T | 0.5 | 0.05638 | 0.01 | 9.52E-09 |
| rs7701440 | C | T | 0.5 | 0.072571 | 0.01 | 3.72E-14 |
| rs783540 | G | A | 0.5 | 0.058689 | 0.01 | 8.45E-10 |
| rs7893279 | T | G | 0.5 | 0.111541 | 0.015 | 4.80E-13 |
| rs7951870 | C | T | 0.5 | 0.093212 | 0.013 | 2.99E-13 |
| rs893949 | C | T | 0.5 | 0.053541 | 0.01 | 2.98E-08 |
| rs9545047 | A | C | 0.5 | 0.05638 | 0.01 | 1.15E-08 |

**Table S11. 19 valid IVs used for MR analysis of neuroticism on IAs.**

| SNP | Effect allele | Non-effect allele | Effect allele frequency | Beta | SE | *P* |
| --- | --- | --- | --- | --- | --- | --- |
| rs10244364 | C | T | 0.3144 | 0.018596 | 0.00302 | 7.35E-10 |
| rs10434704 | C | T | 0.5325 | 0.015905 | 0.002788 | 1.16E-08 |
| rs11082011 | C | T | 0.3342 | 0.020743 | 0.002972 | 2.99E-12 |
| rs12903563 | T | C | 0.4818 | 0.016419 | 0.002869 | 1.04E-08 |
| rs1400867 | G | A | 0.8775 | 0.024964 | 0.004259 | 4.59E-09 |
| rs1690816 | C | T | 0.3134 | 0.017772 | 0.002996 | 2.98E-09 |
| rs17828731 | T | C | 0.5545 | 0.017646 | 0.002799 | 2.87E-10 |
| rs1806153 | T | G | 0.227 | 0.019586 | 0.003404 | 8.77E-09 |
| rs2149351 | T | G | 0.2415 | 0.019342 | 0.003251 | 2.68E-09 |
| rs2273085 | T | C | 0.291 | 0.018494 | 0.003134 | 3.62E-09 |
| rs2359239 | C | T | 0.6144 | 0.01664 | 0.002855 | 5.59E-09 |
| rs2451500 | C | T | 0.3465 | 0.019092 | 0.00292 | 6.23E-11 |
| rs2717036 | C | T | 0.5992 | 0.018566 | 0.002838 | 6.11E-11 |
| rs35991856 | A | C | 0.1328 | 0.02662 | 0.0041 | 8.42E-11 |
| rs4938021 | T | C | 0.6202 | 0.020926 | 0.002921 | 7.86E-13 |
| rs589249 | G | A | 0.3094 | 0.019225 | 0.003056 | 3.17E-10 |
| rs703410 | G | A | 0.7475 | 0.018076 | 0.003229 | 2.17E-08 |
| rs7107356 | G | A | 0.5067 | 0.016127 | 0.002779 | 6.53E-09 |
| rs8084351 | A | G | 0.5089 | 0.021043 | 0.002848 | 1.47E-13 |

**Table S12. 11 valid IVs used for MR analysis of IAs on insomnia.**

| SNP | Effect allele | Non-effect allele | Effect allele frequency | Beta | SE | *P* |
| --- | --- | --- | --- | --- | --- | --- |
| rs10519203 | G | A | 0.371 | 0.12 | 0.02 | 1.29E-09 |
| rs11646044 | G | T | 0.446 | 0.149 | 0.023 | 5.21E-11 |
| rs11661542 | C | A | 0.484 | 0.166 | 0.021 | 5.74E-16 |
| rs12310399 | C | T | 0.354 | 0.138 | 0.02 | 3.25E-12 |
| rs1537373 | G | T | 0.486 | 0.186 | 0.019 | 2.60E-22 |
| rs3742321 | C | T | 0.236 | 0.148 | 0.022 | 4.10E-11 |
| rs39713 | T | C | 0.088 | 0.182 | 0.033 | 4.10E-08 |
| rs4705938 | T | C | 0.549 | 0.12 | 0.019 | 2.55E-10 |
| rs55965782 | T | C | 0.87 | 0.264 | 0.031 | 9.03E-18 |
| rs6997005 | A | G | 0.414 | 0.149 | 0.019 | 1.26E-14 |
| rs79780963 | C | T | 0.922 | 0.225 | 0.039 | 6.82E-09 |

**Table S13. 11 valid IVs used for MR analysis of IAs on major depressive disorder.**

| SNP | Effect allele | Non-effect allele | Effect allele frequency | Beta | SE | *P* |
| --- | --- | --- | --- | --- | --- | --- |
| rs10519203 | G | A | 0.371 | 0.12 | 0.02 | 1.29E-09 |
| rs11646044 | G | T | 0.446 | 0.149 | 0.023 | 5.21E-11 |
| rs11661542 | C | A | 0.484 | 0.166 | 0.021 | 5.74E-16 |
| rs12310399 | C | T | 0.354 | 0.138 | 0.02 | 3.25E-12 |
| rs1537373 | G | T | 0.486 | 0.186 | 0.019 | 2.60E-22 |
| rs3742321 | C | T | 0.236 | 0.148 | 0.022 | 4.10E-11 |
| rs39713 | T | C | 0.088 | 0.182 | 0.033 | 4.10E-08 |
| rs4705938 | T | C | 0.549 | 0.12 | 0.019 | 2.55E-10 |
| rs55965782 | T | C | 0.87 | 0.264 | 0.031 | 9.03E-18 |
| rs6997005 | A | G | 0.414 | 0.149 | 0.019 | 1.26E-14 |
| rs79780963 | C | T | 0.922 | 0.225 | 0.039 | 6.82E-09 |

**Table S14. 8 valid IVs used for MR analysis of IAs on subjective wellbeing.**

| SNP | Effect allele | Non-effect allele | Effect allele frequency | Beta | SE | *P* |
| --- | --- | --- | --- | --- | --- | --- |
| rs10519203 | G | A | 0.371 | 0.12 | 0.02 | 1.29E-09 |
| rs11661542 | C | A | 0.484 | 0.166 | 0.021 | 5.74E-16 |
| rs12310399 | C | T | 0.354 | 0.138 | 0.02 | 3.25E-12 |
| rs1537373 | G | T | 0.486 | 0.186 | 0.019 | 2.60E-22 |
| rs3742321 | C | T | 0.236 | 0.148 | 0.022 | 4.10E-11 |
| rs39713 | T | C | 0.088 | 0.182 | 0.033 | 4.10E-08 |
| rs4705938 | T | C | 0.549 | 0.12 | 0.019 | 2.55E-10 |
| rs6997005 | A | G | 0.414 | 0.149 | 0.019 | 1.26E-14 |

**Table S15.** **10 valid IVs used for MR analysis of IAs on attention deficit/hyperactivity disorder.**

| SNP | Effect allele | Non-effect allele | Effect allele frequency | Beta | SE | *P* |
| --- | --- | --- | --- | --- | --- | --- |
| rs10519203 | G | A | 0.371 | 0.12 | 0.02 | 1.29E-09 |
| rs11646044 | G | T | 0.446 | 0.149 | 0.023 | 5.21E-11 |
| rs11661542 | C | A | 0.484 | 0.166 | 0.021 | 5.74E-16 |
| rs12310399 | C | T | 0.354 | 0.138 | 0.02 | 3.25E-12 |
| rs1537373 | G | T | 0.486 | 0.186 | 0.019 | 2.60E-22 |
| rs3742321 | C | T | 0.236 | 0.148 | 0.022 | 4.10E-11 |
| rs39713 | T | C | 0.088 | 0.182 | 0.033 | 4.10E-08 |
| rs4705938 | T | C | 0.549 | 0.12 | 0.019 | 2.55E-10 |
| rs55965782 | T | C | 0.87 | 0.264 | 0.031 | 9.03E-18 |
| rs6997005 | A | G | 0.414 | 0.149 | 0.019 | 1.26E-14 |

**Table S16. 10 valid IVs used for MR analysis of IAs on autism spectrum disorder.**

| SNP | Effect allele | Non-effect allele | Effect allele frequency | Beta | SE | *P* |
| --- | --- | --- | --- | --- | --- | --- |
| rs10519203 | G | A | 0.371 | 0.12 | 0.02 | 1.29E-09 |
| rs11646044 | G | T | 0.446 | 0.149 | 0.023 | 5.21E-11 |
| rs11661542 | C | A | 0.484 | 0.166 | 0.021 | 5.74E-16 |
| rs12310399 | C | T | 0.354 | 0.138 | 0.02 | 3.25E-12 |
| rs1537373 | G | T | 0.486 | 0.186 | 0.019 | 2.60E-22 |
| rs3742321 | C | T | 0.236 | 0.148 | 0.022 | 4.10E-11 |
| rs39713 | T | C | 0.088 | 0.182 | 0.033 | 4.10E-08 |
| rs4705938 | T | C | 0.549 | 0.12 | 0.019 | 2.55E-10 |
| rs55965782 | T | C | 0.87 | 0.264 | 0.031 | 9.03E-18 |
| rs6997005 | A | G | 0.414 | 0.149 | 0.019 | 1.26E-14 |

**Table S17. 10 valid IVs used for MR analysis of IAs on bipolar disorder.**

| SNP | Effect allele | Non-effect allele | Effect allele frequency | Beta | SE | *P* |
| --- | --- | --- | --- | --- | --- | --- |
| rs10519203 | G | A | 0.371 | 0.12 | 0.02 | 1.29E-09 |
| rs11646044 | G | T | 0.446 | 0.149 | 0.023 | 5.21E-11 |
| rs11661542 | C | A | 0.484 | 0.166 | 0.021 | 5.74E-16 |
| rs12310399 | C | T | 0.354 | 0.138 | 0.02 | 3.25E-12 |
| rs1537373 | G | T | 0.486 | 0.186 | 0.019 | 2.60E-22 |
| rs3742321 | C | T | 0.236 | 0.148 | 0.022 | 4.10E-11 |
| rs39713 | T | C | 0.088 | 0.182 | 0.033 | 4.10E-08 |
| rs4705938 | T | C | 0.549 | 0.12 | 0.019 | 2.55E-10 |
| rs55965782 | T | C | 0.87 | 0.264 | 0.031 | 9.03E-18 |
| rs6997005 | A | G | 0.414 | 0.149 | 0.019 | 1.26E-14 |

**Table S18. 9 valid IVs used for MR analysis of IAs on schizophrenia.**

| SNP | Effect allele | Non-effect allele | Effect allele frequency | Beta | SE | *P* |
| --- | --- | --- | --- | --- | --- | --- |
| rs11646044 | G | T | 0.446 | 0.149 | 0.023 | 5.21E-11 |
| rs11661542 | C | A | 0.484 | 0.166 | 0.021 | 5.74E-16 |
| rs12310399 | C | T | 0.354 | 0.138 | 0.02 | 3.25E-12 |
| rs1537373 | G | T | 0.486 | 0.186 | 0.019 | 2.60E-22 |
| rs3742321 | C | T | 0.236 | 0.148 | 0.022 | 4.10E-11 |
| rs39713 | T | C | 0.088 | 0.182 | 0.033 | 4.10E-08 |
| rs4705938 | T | C | 0.549 | 0.12 | 0.019 | 2.55E-10 |
| rs55965782 | T | C | 0.87 | 0.264 | 0.031 | 9.03E-18 |
| rs6997005 | A | G | 0.414 | 0.149 | 0.019 | 1.26E-14 |

**Table S19. 11 valid IVs used for MR analysis of IAs on neuroticism.**

| SNP | Effect allele | Non-effect allele | Effect allele frequency | Beta | SE | *P* |
| --- | --- | --- | --- | --- | --- | --- |
| rs10519203 | G | A | 0.371 | 0.12 | 0.02 | 1.29E-09 |
| rs11646044 | G | T | 0.446 | 0.149 | 0.023 | 5.21E-11 |
| rs11661542 | C | A | 0.484 | 0.166 | 0.021 | 5.74E-16 |
| rs12310399 | C | T | 0.354 | 0.138 | 0.02 | 3.25E-12 |
| rs1537373 | G | T | 0.486 | 0.186 | 0.019 | 2.60E-22 |
| rs3742321 | C | T | 0.236 | 0.148 | 0.022 | 4.10E-11 |
| rs39713 | T | C | 0.088 | 0.182 | 0.033 | 4.10E-08 |
| rs4705938 | T | C | 0.549 | 0.12 | 0.019 | 2.55E-10 |
| rs55965782 | T | C | 0.87 | 0.264 | 0.031 | 9.03E-18 |
| rs6997005 | A | G | 0.414 | 0.149 | 0.019 | 1.26E-14 |
| rs79780963 | C | T | 0.922 | 0.225 | 0.039 | 6.82E-09 |
