# Supplementary material for: Investigating Causal Relationships Between Psychiatric Traits and Intracranial Aneurysms: A Bi-directional Two-Sample Mendelian Randomization Study
Source: Front Genet. 2021 Oct 19;12:741429. doi: 10.3389/fgene.2021.741429 (PMC8560679; doi:10.3389/fgene.2021.741429)
Supplement: Supplementary file 2 [file DataSheet1.docx]

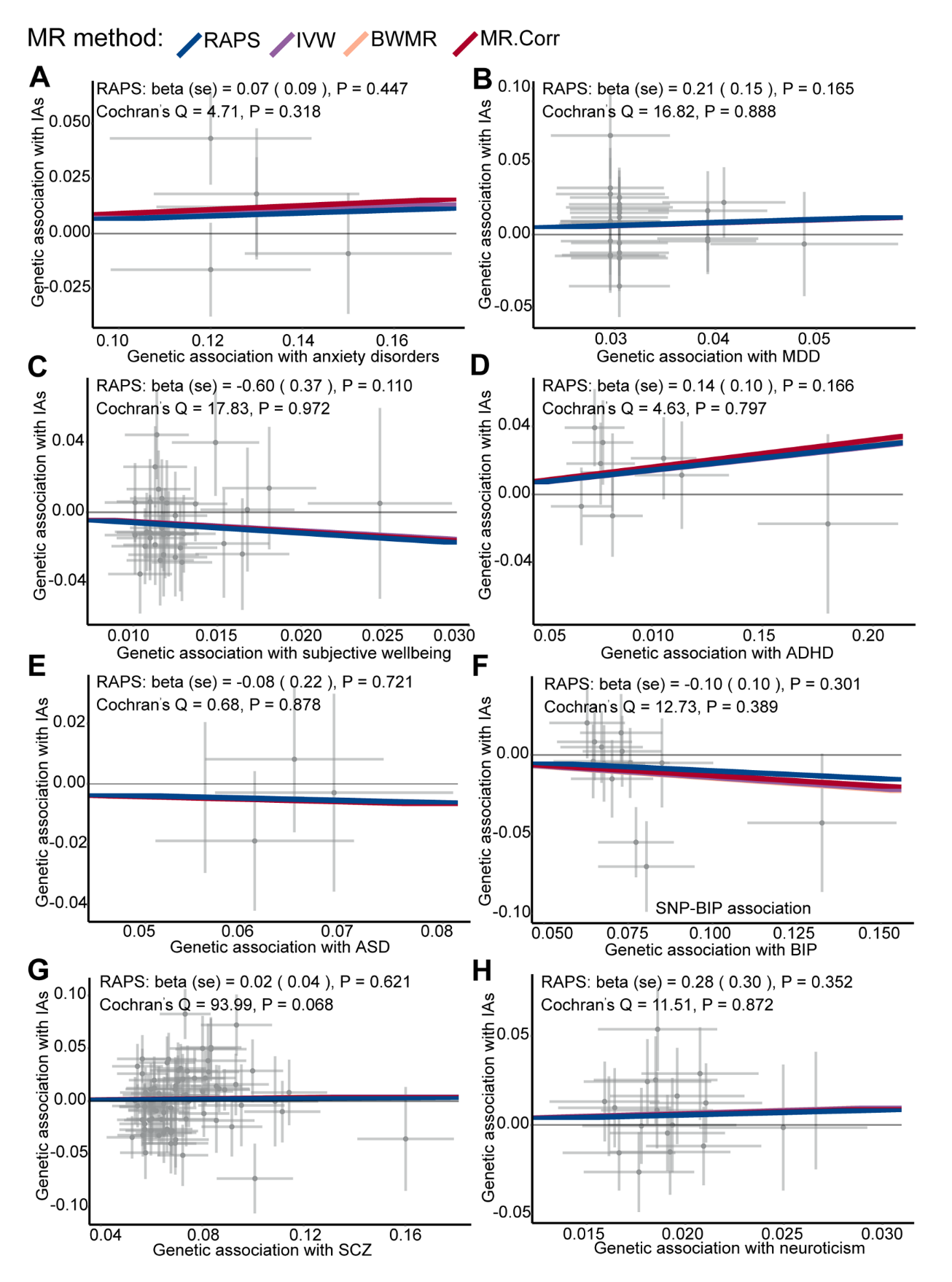


**Figure S1** Scatter plots of SNPs with IAs versus. SNPs with anxiety disorders (**A**), MDD (**B**), subjective wellbeing (**C**), ADHD (**D**), ASD (**E**), BIP (**F**), SCZ (**G**), and neuroticism (**H**) for all the valid IVs. Each dot represents one SNP, with corresponding standard error bars of its relation to psychiatric traits (x-axis) and IAs (y-axis); the colored solid lines represent estimated causal effect values of four MR methods.


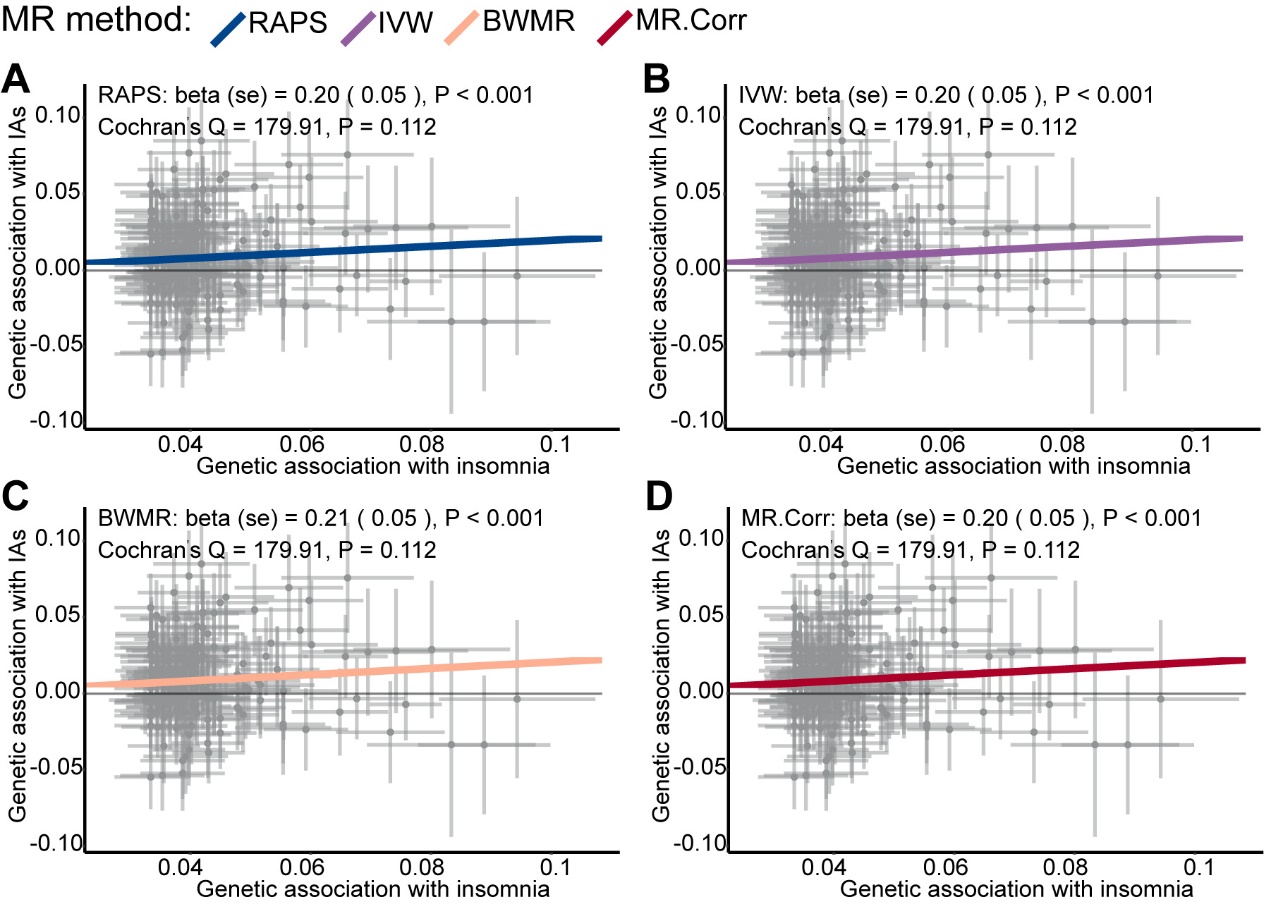


**Figure S2** Four MR methods (scatter plots) of SNPs with IAs versus. SNPs with insomnia for all the valid IVs. The colored solid lines represent estimated causal effect values of four MR methods: RAPS (**A**), IVW (**B**), BWMR (**C**), MR.Corr (**D**). Each dot represents one SNP, with corresponding standard error bars of its relation to psychiatric traits (x-axis) and IAs (y-axis).


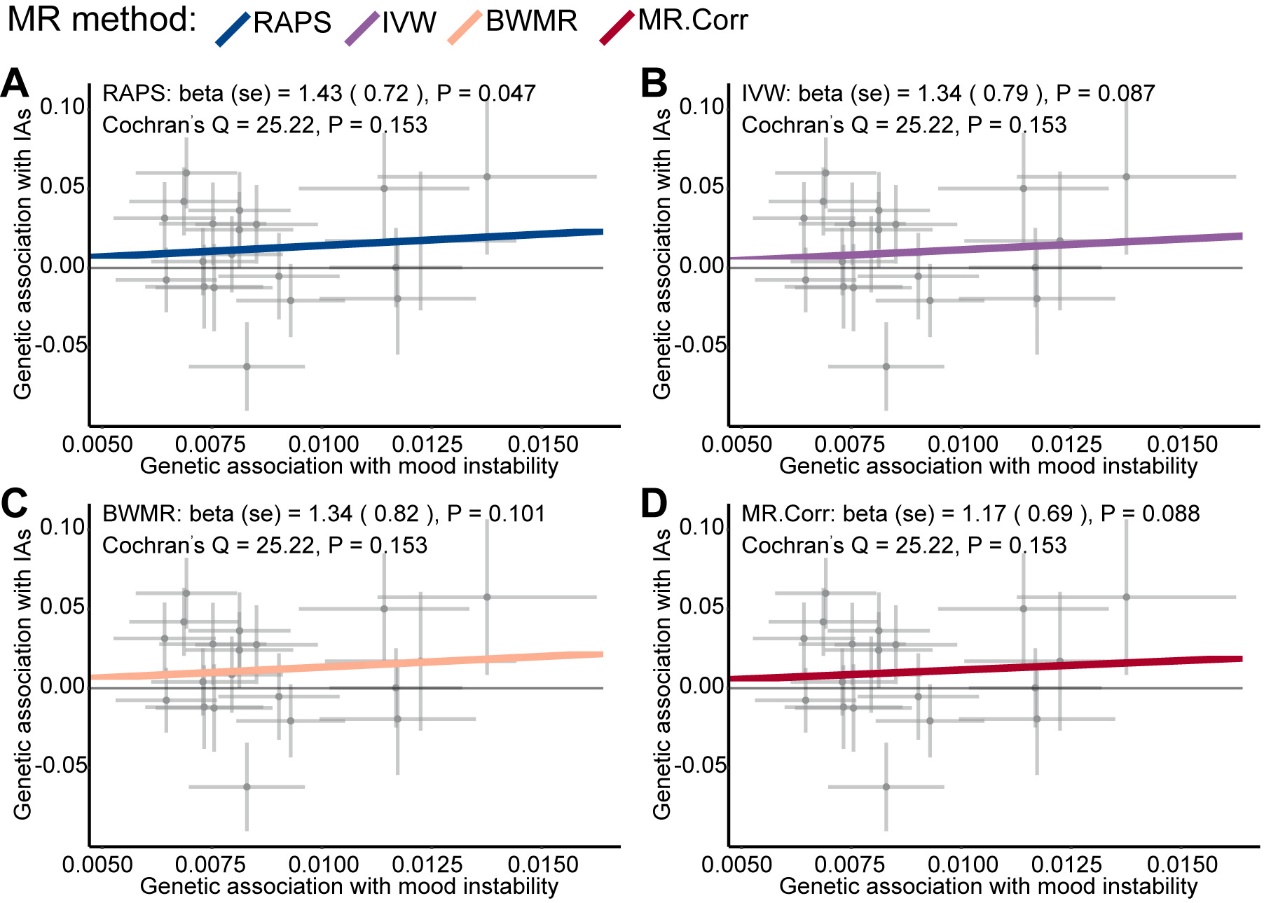


**Figure S3** Four MR methods (scatter plots) of SNPs with IAs versus. SNPs with mood instability for all the valid IVs. The colored solid lines represent estimated causal effect values of four MR methods: RAPS (**A**), IVW (**B**), BWMR (**C**), MR.Corr (**D**). Each dot represents one SNP, with corresponding standard error bars of its relation to psychiatric traits (x-axis) and IAs (y-axis).


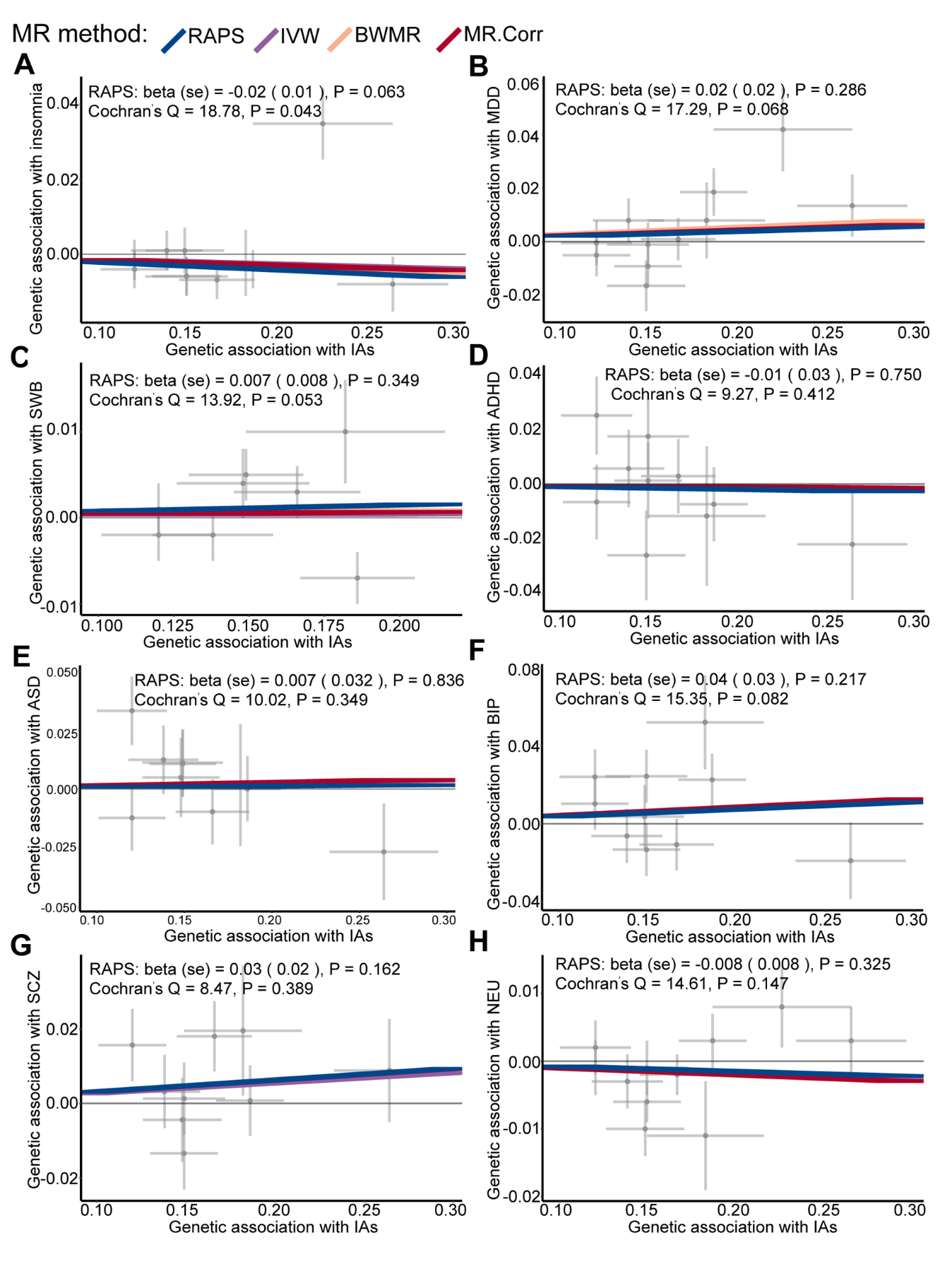


**Figure S4** Scatter plots of SNPs with eight psychiatric traits versus. SNPs with IAs

Insomnia (**A**), MDD (**B**), subjective wellbeing (**C**), ADHD (**D**), ASD (**E**), BIP (**F**), SCZ (**G**), neuroticism (**H**). Each dot represents one SNP, with corresponding standard error bars of its relation to psychiatric traits (y-axis) and IAs (x-axis); the colored solid lines represent estimated causal effect values of four MR methods.
